# A human centred approach to digital technologies in health care delivery among mothers, children and adolescents

**DOI:** 10.1186/s12913-022-08744-2

**Published:** 2022-11-23

**Authors:** Yasini Haroun, Richard Sambaiga, Nandini Sarkar, Ntuli A. Kapologwe, James Kengia, Jafary Liana, Suleiman Kimatta, Johanita James, Vendelin Simon, Fatma Hassan, Romuald Mbwasi, Khadija Fumbwe, Rebecca Litner, Gloria Kahamba, Angel Dillip

**Affiliations:** 1 Department of Sociology and Anthropology, University of Dar es Salaam, Dar es Salaam, Tanzania; 2 Institute of Tropical Medicine, Antwerp, Belgium; 3 Director of Health Services, The President’s Office-Regional Administration and Local Government (Po-RALG), Dodoma, Tanzania; 4 The President’s Office-Regional Administration and Local Government (Po-RALG), Coordinator Regional Health Management Teams, Tanzania,, Tanzania; 5 Apotheker Consultancy (T) Limited, Dar es Salaam, Tanzania; 6 D-tree International, Dar es Salaam, Tanzania

**Keywords:** Maternal health, Healthcare delivery, CHWs, ADDOs, Health facilities, Human centred, Digital intervention, Afya-Tek

## Abstract

**Background:**

Healthcare outcomes in child, adolescent and maternal in Tanzania are poor, and mostly characterised by fragmentary service provision. In order to address this weakness, digital technologies are sought to be integrated in the milieu of health as they present vast opportunities especially in the ability to improve health information management and coordination. Prior to the design and implementation of the Afya-Tek digital intervention, formative research was carried out to ensure that the solution meets the needs of the users. The formative research aimed to examine: the burden of disease and related health seeking behaviour; workflow procedures and challenges experiencing healthcare actors; adolescent health and health seeking behaviour; and lastly examine technological literacy and perceptions on the use of digital technologies in healthcare delivery. This paper therefore, presents findings from the formative research.

**Methods:**

The study employed exploratory design grounded in a qualitative approach. In-depth interview, focus group discussion, participant observation and documentary review methods were used for collecting data at different levels. The analysis was done thematically, whereby meaning was deduced behind the words which the participants used.

**Results:**

Findings suggest that the perceived burden of diseases and health seeking behaviour differ across age and social group. Multiple work-related challenges, such as lack of proper mechanism to track referrals and patient’s information were noted across healthcare actors. There was a keen interest in the use of technologies shown by all study participants to improve care coordination and health outcomes among health system actors. Participants shared their views on how they envision the digital system working.

**Conclusion:**

The formative research provided insightful background information with regard to the study objectives. The findings are used for informing the subsequent phases of the co-development and implementation of the Afya-Tek digital health intervention; with a view to making it relevant to the needs of those who will use it in the future. As such, the findings have to a large extent met the purpose of the current study by envisaging the best ways to design digital intervention tailored to meet the needs of those who will be using it.

## Background

High prevalence of health problems related to mothers and new-borns constitutes a global public health concern [1, 2]. Evidence from previous research indicates that children face the risk of death in their first 28 days. The World Health Organization report of 2020 shows that 2.4 million children died in the first month of life in 2019 globally, which is approximately 6700 new-born deaths everyday [1]. Preterm birth, intrapartum-related complications, infections and birth defects are acknowledged to amount to increasing numbers of neonatal deaths [2]. Poor quality health care is also cited to be the main contributor to the early deaths [3].

Additionally, maternal mortality has continued to jeopardise the lives of women globally. It was reported in 2017, for instance, that approximately 810 women died everyday due to causes associated with pregnancy and childbirth complications [1, 4]. Adolescents face a higher risk of complications and deaths linked to pregnancies than other women [2]. Moreover, between 80 and 90% of the deaths occur in low and middle-income countries [3, 5]. In Tanzania, more than 600,000 children under-five die of preventable causes, more than 1.2 million women experience unintended pregnancies (including 26% of adolescent girls aged 15–19), and over 9,000 women die of preventable maternal complications each year [1, 4].

A significant contributor to the health indicators is poor quality healthcare within the Tanzanian primary health system. The five-star rating system assessment in 2016/2017 indicated in 7,000 health facilities across Tanzania, a mere two (2) percent of facilities met the minimum standard of quality of healthcare (three stars or more), and 34% received a zero star, suggesting that they were providing extremely sub-optimal levels of care. The assessment system considered management of health facility and staff performance, accountable and respectful care, safety and cleanliness, and quality of care [2].

The global community recognizes the importance of task shifting as a strategy to bring preventive and curative care closer to communities, reducing the workload of facility health staff, providing a people-centred continuum of care, and strengthening the overall health system are crucial in fighting the rising number of deaths [3]. It is estimated that 50% of child deaths occur outside the formal health sector, most of which occurring at home among children who have not been assessed by a health worker. In Tanzania, there are two cadres of health workers are supporting individuals at the community level: Community Health Workers (CHWs) and Accredited Drug Dispensing Outlets (ADDOs) (5).

Community Health Workers (CHWs) initiate and manage referrals of individuals from the community to the primary health facility, however the tracking of these referrals remains a challenge. In most of African countries, the paper-based system and distance to health facilities can be a significant barrier to healthcare seeking [6]. A study in Uganda found that 72% of referrals from the community to a health facility are never completed, which is consistent with partner experiences working with CHWs in Tanzania [7]. Among others, CHWs’ lack of motivation and other work-related challenges such as poor linkage among healthcare actors contribute to incompleteness of the referrals.

ADDOs, private drug outlets whose role is to manage minor illness and dispense medicines, are not formally certified to examine and diagnose clients. ADDO dispensers do not have clinical guidance for decision-making processes on how to manage sick clients who often come with a wide variety of symptoms. With their limited training, they use their judgement when assessing clients to determine whether to refer to a health facility, provide treatment, calculate the correct dosage, and provide follow-up advice [5].

With the proliferation of digital platforms, digital innovation and technologies present vast opportunities in healthcare delivery, especially in their ability to improve health information management and coordination among health care providers. Afya-Tek, a digital health intervention, linking CHWs, ADDOs and health facilities for referral tracking and care coordination, aims at improving maternal, child and adolescent outcomes in Tanzania. Prior to designing and implementing the Afya-Tek intervention, formative research was carried out in the districts of the Coast regions; Kibaha District Council and Kibaha Town Council as a first phase of work, which was essential to guide development of the digital intervention.

The main objectives of the formative research were to: understand the perceived burden of disease and related health seeking behaviour; identify CHWs and ADDO workflow procedures and challenges experienced by CHWs, ADDOs and health facilities in care coordination; investigate adolescent health and health seeking behaviour; and lastly examine technological literacy and perceptions on the use of digital technologies in healthcare delivery. The formative research findings will be used for informing the next phases of the Afya-Tek project in the co-development of digital health intervention and to ensure that the solution is designed specifically to meet the needs of those who will be using the technology.

## Afya-Tek program

Afya-Tek is a digitally enabled, community based responsive health system initiative that brings together the key health actors at the community level. The project links Community Health Volunteers (CHWs), Accredited Drug Dispensing Outlets (ADDOs), and public health facilities. The linkage aims to improve decision making, prompt access to care through referrals and overall quality of care along the continuum of care. By using the digital platform recommended by the Government of Tanzania (Open Smart Register Platform), these key players will be empowered to efficiently refer patients to the appropriate service, track their status, and follow up to ensure proper and respectful care.

Moreover, decision support tools (tailored to Government-approved screening protocols for danger) will be used by CHWs and ADDO dispensers to screen patients and refer them to appropriate care. Unique biometric identification through fingerprint scanning will enable the digital system to not only increase efficiency and consistency of treatment through patient tracking, but it will also prevent valuable patient information from being lost or unnecessarily duplicated. The goal is to save lives and improve health and wellbeing of children, mothers, adolescents, and their families through a digitally-enabled continuum of care that can be scaled at a sustainable cost, while creating substantial learning about how emerging technologies can be combined to amplify program effectiveness.

The Afya-Tek initiative is guided by three approaches: (a) human-centred participatory design necessary to inform the design of the Afya-Tek digital intervention; (b) digital health; and (c) biometric identification. The human-centred design approaches include the formative and embedded development and research strategies, as well as realist monitoring and evaluation processes [8, 9]. All stages of the process are co-created and co-produced by the local communities, health systems actors, app-developers, researchers, and implementers. This is necessary for ensuring that the digital intervention and all associated research are closely aligned to local needs and resources.

The current formative research acts as a precursor to the co-development of the digital system. By understanding barriers to and facilitators of continuity of care in the formal health system—including current care-seeking beliefs and behaviour of community members, as well as the practices, motivations and challenges of health workers, the Afya-Tek program seeks ways within which it will improve the continuum of care. Following this, through community participatory approaches, the Afya-Tek program will promote co-creation of the digital health innovation with all relevant stakeholders in the study districts. This aims at ensuring the solution is designed specifically to meet the needs of those who will be using the technology and the larger health system. A key aspect of this process will be to understand how digital tools will improve the work of each of the three cadres.

The program will also use Realist Evaluation [9], a participatory theory-driven evaluation approach, in which we shall be asking not just “does the intervention work?”, but more so, “how or why does this digital intervention work, for whom, and in what circumstances? We will analyse, for instance, how and why these digital tools can improve the continuity of referral processes between health providers. How they can boost the confidence and competence of CHWs and health workers through decision support, or help in automating government reporting, or improve connectedness, supervision and mentorship within the health system through data sharing. Learning how and why the intervention works at all possible levels of its influence can provide answers to how best to adapt the intervention to new contexts, thereby promoting the future possibility of scaling up the digital initiative.

This paper presents findings of the formative research of the Afya-Tek program conducted in the two district councils in the Coast Region as a first phase of work, which was essential to guide development of the digital intervention.

## Methodology

### Study design

We conducted a qualitative exploratory study. We employed qualitative methods of data collection such as in-depth interviews, focus group discussion, participant observation, and documentary review. The study was conducted from November 2019 to February 2020. The design offered an opportunity to gain a deeper understanding of required aspects of the community and health practices and experiences prior to the co-development of the digital health intervention. The study was inspired by the principles of grounded theory [10] as such the study was iterative in nature. Continuous field data analysis and interpretation provided insights used to revise the interview guides on an ongoing basis.

### Setting of the study

The Afya Tek project operates in two administrative district councils of Kibaha District in the Coast Region. Administratively, Kibaha is divided into two councils: Kibaha District Council (DC) and Kibaha Town Council (TC). Kibaha is bordered to the North by the Bagamoyo District, to the South by Kisarawe District, to the East by Dar es Salaam, and to the West by Morogoro Region. It is directly linked with Bagamoyo town by seasonal road, while connected to other district headquarters such as Kisarawe, Mkuranga, Kilindi and Utete (Rufiji).

The 2012 census conducted indicated that the total population of Kibaha amounts to 200,000 people. Kibaha is inhabited by people from different ethnic groups and cultural diversities, including religion. The main ethnic groups found in the district include Zaramo, Kwere, Doe, Masai, and Barbaig. Kibaha has a vast area of land that allows people to engage in various economic activities, including agriculture, livestock keeping, beekeeping, trade, processing (timber, flour mills) and small-scale entrepreneurship. This study is part of the larger Afya Tek project which is being implemented in the two councils in Kibaha.

### Sampling and recruitment of study participants

The research participants were purposively sampled to ensure that relevant information as per the study objectives is obtained. The sampling strategy based on the principles of gradual selection, as well as maximal variation to capture prospective differences in responses between the participant groups [11]. Our sampling based on saturation principles, that is, we continued sampling new participants until no new information emerged from the responses [12]. The recruitment of participants was conducted through the existing community-based networks in Kibaha TC and DC. Responsible district councils’ authorities in Kibaha TC and Kibaha DC were aware of the study, and supported us by ensuring a successful data collection exercise. Data were collected at different levels of potential users of the Afya-Tek intervention, beneficiaries and other relevant stakeholders in the two Councils (Table 1).

**Table 1:** Participants in the study

	Study participants and their professional background	Methods of data collection	
		**In-depth Interviews**	**Focus group discussions**
**District Level**	District Pharmacist	2	-
	Data Manager	2	-
	District Community Health Worker’s Coordinator	1	-
**Village & Ward Level**	Religious Leaders	6	-
	Traditional Birth Attendants	4	-
	Herbalists	4	-
	Traditional Healers	4	-
	Village Executive Officers	1	-
	Local Government Leaders	-	2
**End-users Level**	Community Health Volunteers	63	2
	ADDO dispensers/Owners	32	-
	Health Facility/Dispensary Workers	43	-
**Beneficiary Level**	Adult Female	13	7
	Adolescent Girls	20	8
	Adolescent Boys	20	7
**Total**		215	26

### Demographic composition of the participants

In this section we are describing the demographic information of the participants. Religion wise, most of the participants were Muslims. It should be noted that Kibaha is one of the coastal towns where Islamic religion is dominant. Regarding the level of education, there were variations between the groups; most of CHWs, both adult males and females, aged 27–50 years, had completed secondary education. In terms of occupation, apart from their formal CHW work, most CHWs were also farmers and entrepreneurs.

Healthcare providers comprised registered nurses, assistant medical officers, medical doctors, CHW supervisors, reproductive and child health staff, clinical officers, data managers, and CHMT members. Health providers’ age range was 35–48 years. Majority of the ADDO dispensers were women, and also worked as nurses in health facilities, farmers and entrepreneurs. Most of out of school adolescent girls, aged 15–19 years, had children. They are engaging in small-scale entrepreneurship jobs like selling buns, fish and other food stuffs. Majority of them had completed primary education while others had dropped out of secondary school because of pregnancy. Adolescent boys, aged 15–19 years, who were part of the study were either in secondary school or out of school.

### Data collection procedures

14 experienced research assistants were recruited and trained by the research team, using the developed preliminary Swahili interview guides. The data were collected in Swahili language by a team of trained researchers and research assistants. We collected primary data through qualitative (in-depth) interviewing method, focus group discussions, and participant observations. The rationale for using these methods was based on the reality that understanding background information in relation to the study objectives prior to project design and implementation required in-depth exploration of first-hand accounts and behavior of the participants.

One to one form of interviews was carried out among three groups of participants (ADDO dispensers, health facility staff, and district officials) at their workplaces. Community Health Workers were interviewed at their convenient locations within the community, which included health facilities and village offices. The interview process was flexible enough to allow ADDO clients/fellow staff to access services in-between. Healthcare providers at health facilities were interviewed at their respective facilities when they had free time. Patients and community members were interviewed at their homes or in other private locations as per their preference. At the national level, in-depth interviews were conducted with the Pharmacy Council Registrar and officials from PO-RALG. The length of each in-depth interview would take between 45 and 60 min.

Focus group discussions were held with maternal, pregnant women, adolescents, CHWs and CHMTs at different places including village/ward offices, health facilities, and schools. Focus group discussions followed key interviews (in-depth interviews) after identifying knowledgeable individuals to hold group discussions. Focus group discussions were employed to gain social groups general consensus and mapping predominant social ideals on issues under study. The length of each FGD would take between 60 and 90 min depending on the activeness of the participants and emerging issues under discussion. All the in-depth interviews and focus group discussions were recorded with audio recording devices but at the same time, research assistants also took notes of the key issues raised during the sessions.

A total of 30 participant observations were conducted in the CHW, ADDO, and health facility settings; For easy management of observations and cases, 5 observations per group of stakeholders (CHWs, ADDOs and health facilities) were carried out from each of the two councils. This involved taking part in daily routine of healthcare seekers and deliverers to further explore health seeking and delivery behaviours among patients and caregivers. It also involved extensive engagement in community health activities to further explore patient interaction, use of referral forms and patient registers at both CHW and ADDO levels. At the health facility, the system of receiving patients was observed, along with patient files and registers, which are all currently paper based.

### Data management and analysis

The interview data generated from IDIs and FGDs were in the form of personal handwritten notes based on researcher memos, and the digital audio-recordings. The observational data was recorded through diary and fieldnotes. The primary data were then converted into written electronic form and transferred into computer databases. All digital recordings of IDIs and FGDs were transcribed verbatim in Swahili, and where needed translated into English. Overall data processing and organization was managed using the NVivo QSR (Version 12+). This computer assisted qualitative data analysis software helped sort and organize the bulk of raw data generated.

Thereafter, data was transformed into meaningful findings. This required engaging thematic analytic procedure to analyse the data in line with research questions. The data was deduced behind the words which the participants used. Code frames were developed to generate themes. Coding was done by four different research assistants before final agreement on the final codes to ensure consistency and agreement. This list of thematic codes was reviewed and grouped into categories and themes for analysis by the team of four researchers. Analysis was undertaken using a framework of grouping relevant themes that answer key issues as per the study objectives. This was then used to facilitate the analysis of thematic concerns and trends arising from the narratives.

### Ethical consideration

Ethical clearance was sought and obtained from the National Institute for Medical Research of Tanzania (NIMR) and the IRB of Institute of Tropical Medicine, Antwerp. An information sheet about the study was written in Swahili, explaining why it is being carried out, by who, what it will involve, as well as the rights of the participants. During recruitment and prior to participation, informed consent was sought from all participants by a written form. For adolescents who were under the age of 18 years, consent was sought from their parents or guardians, while for those adolescents and remaining participants aged 18 years and above, the consent was given by themselves. Among the key concerns taken into consideration was to safeguard the dignity, rights as well as safety of the participants. Confidentiality of all study participants was assured through practices of pseudonymisation. For the purpose of ensuring confidentiality, all data presentation has used pseudo names.

## Results

### Perceived burden of diseases and related health seeking behaviour

#### Perceived burden of disease

The burden of disease was differentiated by social group and age of people. Health problems reported to face children under the age of five include most of the infectious diseases and conditions like diarrhoea, Malaria, Urinary Tract Infection (UTI), pneumonia, worm infections, malnutrition, asthma, convulsion, umbilical cord infections, skin rashes, stomach fever, and respiratory infections. While the majority of participants associated such illnesses and diseases with natural causes, others believed ‘evil eye’ by jealous and neighbours to contribute towards children’s diseases. This was noted to be a common belief particularly in the coastal regions, Kibaha being one, children are always not exposed to people because of superstition beliefs.

Among pregnant women, reported common illnesses and diseases include anaemia, foot swelling, back pain, blood pressure, malaria, UTI, itching in genital areas, epilepsy, headache, and bleeding. Many of the participants thought that diseases facing pregnant women are partly caused by delays in clinic visits by pregnant women themselves, and not following medical advice. It was also reported that the majority of pregnant women do not attend clinic during the first trimester. Delays in or not following medical advice was contributed by several factors, but not limited to; a deep-seated tendency of many pregnant women to attend clinic when the health status is severe and lack of prompt care in some of the care stations. The statement below illustrates what is maintained by one of the participants:

*‘‘Most of these problems are due to the fact that women attended clinics too late. Not all attend their compulsory four visits, at least now the percentage is increasing because of the intervention, but still more needs to be done.’* (CHMT, Kibaha DC).

*‘‘There are different reasons why women delay attending clinics and not follow medical advice. You know, in this community people have a tendency to seek care when diseases or health conditions are severe. Others may start by assessing the kind of care they get and decide not to follow what is advised by the medical advisers. We need to continue educating them to attend clinics and follow medical advice accordingly.’’* (Health Facility staff, Kibaha DC).

Perceived diseases and illnesses among postpartum mothers include dizziness and frequent bleeding, fistula, UTI, foot pain, anaemia caused by excessive bleeding, back pain, and periodic fever. As for pregnancy complications, the participants thought that most of the diseases facing women after delivery are caused by women not following medical advice like taking proper diet and seeking prompt care when needed.

Among adolescents, the main reported diseases include UTI and Sexually Transmitted Infections (STIs) such as gonorrhoea and syphilis. In the study area, those who are infected with these conditions are said to be vulnerable to HIV/AIDS. The prevalence of STIs among adolescents as shown in the findings was exacerbated by poor access and multiple barriers to accessing quality reproductive health services.

*‘‘Adolescents as an active sexual group are vulnerable to sexually transmitted infections or diseases. Gonorrhoea and syphilis are the main STIs facing this group. They come here for medication and advice sometimes. The problem is that reproductive health services are sometimes hard to access because adolescents prefer friendly services that are also private’’* (ADDO dispenser, Kibaha DC).

#### Reported community health seeking behaviour

Communities were reported to mostly seek treatment and advice from Accredited Drug Dispensing Outlets (ADDOs) and attend health facilities for serious health problems. Conversely, infants and children under the age of five were taken straight to health facilities when they fall sick. Traditional healers were also an option for treatment by Kibaha communities as elaborated here:

*“Some people think that they are being bewitched; but the truth is, not every disease that one faces is caused by witches. There are those that once they fall sick, they go to traditional healers. We always educate them when they attend health facilities that diseases have their natural causes, and particularly for young children who need to be checked for their health status now and then because of their vulnerability to various illnesses.”* (Health facility staff, Kibaha TC).

It was also reported by the participants that herbalists are also consulted by pregnant women, who visit them for treatment for the purpose of speeding up delivery in case they are due and do not feel any signs of delivery. In the discussion with herbalists, this was narrated:

*“Some pregnant women do not deliver when they are due. They come here while their pregnancies are 14 months* (referring to a 14-month gestation). *You know, pregnant women have this superstition game that they play. A fellow pregnant woman can block your delivery. So, when they come here, I treat them with herbs, and in two days, they feel the labour pain, and deliver.”* (Herbalist, Kibaha DC).

Pregnant women’s consultation with herbalists was also reported by health facility staff in the two study districts as narrated here:

*“When it comes to labour, a lot of women are using local herbs, a woman many with premature labour, after two hours, she experiences intensive labour and the pain that she goes through, you just say no; this is not normal. When you ask her, she says that she has taken herbs to speed up the delivery. Imagine, just recently we had two maternal deaths from taking local herbs.”* (CHMT, Kibaha DC).

Herbalists were also reported to be consulted by breastfeeding mothers for herbs to stimulate milk production. Religious leaders were also mentioned as a treatment resort for community members who believe in faith healing. In conversations with religious leaders, most reported to be followed by mothers whose children are sick, after prayer sessions, religious leaders reported to always tell mothers to take their children to health facilities for further examination.

### Adolescent health and health seeking behaviour

Definition of adolescents varied among study participants. While adolescents were said to start from age 12 to 20, others reported to start from 15 to 35. It was noted that the Kibaha community had their cultural definition of an adolescent. To them, adolescent is any youth that has gone through initiation rituals or what is commonly known in Swahili as ‘unyago’ and ‘jando’ for girls and boys respectively. In Tanzania these initiation rituals are popular cultural practices among coastal people. During *unyago* girls are considered grown-ups and ready to have families. *Unyago* represents itself as an important milestone in a girl’s life. It is perceived to acquaint girls with knowledge and experience of handling sexual related matters and reproduction.

Girls were reported to start sexual practices at the age of 12 while boys at 16. Adolescent pregnancy is a situation regarded normal as long as the girl has gone through *unyago* rituals as narrated here:

*“Mhh! The communities here perceive pregnancy as something normal to them. Once a girl completes primary education, she undergoes the ‘unyago’ ritual. By the time she joins secondary school, she is pregnant already, and this is normal for the community. What is important is that she has passed through the ritual.*” (CHMT, Kibaha TC).

ADDO dispensers opined that, sexual practices among adolescents are influenced by some community members themselves. This is because some of the community members take young children to purchase contraceptives at the outlets as explained here:

*“A young boy may come to the outlets and ask me to give him a condom, but when you look at him, he is not even 10 years old. I have to ask him what he needs a condom for. He replies, “Someone has sent me,”. “I always don’t dispense for such cases,” I tell him go and bring the one who sent him. He comes with a man, and you ask yourself questions: How can this man send a young boy to buy a condom? What does this mean?”* (ADDO dispenser, Kibaha TC).

*‘‘You might be wondering, but the fact is that they get pregnant. Now, you know the problem is that they are school girls, and they wish to continue with their studies. They normally go for abortion sometimes in ADDO shops or health facilities’’* (CHW supervisor, Kibaha Dc).

Adolescent boys and girls were reported to access FP services at ADDOs. ADDOs were mostly preferred because of privacy. The commonly reported health problems facing adolescents include STIs, HIV/ AIDS, UTI, tuberculosis and Malaria. For STIs, adolescents prefer to seek treatment in ADDOs rather than health facilities because they are assured of privacy. Socio-cultural beliefs associated with stigma pose fear among adolescents to access such services. This accelerates their preference for healthcare in ADDOs even when they are located far away from their villages, wards or districts. It was also found that the common methods used for family planning are condoms for boys and pills for girls.

ADDO dispensers frequently receive adolescents in need of family planning education and related services. Adolescents prefer not seeking sexual and reproductive health care in health facilities even when advised to for further examination. Most of the youth refuse and insist to be provided with whatever treatment that is available at the drug outlets, even when the price is high as explained here.

*‘‘Our family planning services are not youth friendly. The building that offers family planning services is obvious, everyone knows it. Young girls are afraid of coming here, otherwise, everyone will know that they are sexually active, and engage in unsafe sex. They do not come here’’* (Health facility staff, Kibaha DC).

Treatment seeking among adolescents was reported to be hindered by some parents of adolescents who refuse to allow health facility staff and CHV to access their children for education on FP, for the reason that children are still young to be educated on reproductive health issues. This was said to contribute to adolescent pregnancies.

### Workflow procedures and challenges experienced by CHWss, ADDOs and health facilities in care coordination

#### Community Health Workers’ Workflow Procedures and Challenges

CHWs reported that their main roles include visiting households and providing health education to household members. As part of education provision, CHWs also advise sick people and those with chronic diseases to attend health facilities for care and treatment. CHWs reported that pregnant women and children are among the most important groups they advise to seek care from health facilities and never to miss the antenatal clinic visits. CHWs reported to plan their own workflow in collaboration with their supervisors at health facilities and household members they visit. For example, they know when they need to work at health facilities and the best time to visit the households. On average, CHWs visit between four and eight households per day, depending on the distance between households. Bicycles were the most used means of transport to reach households.

Most work three to four days per week and use the remaining days for non CHWs related activities. It was the intention of the current study to explore the average time a CHW spends in one household to better inform project intervention activities. The majority reported to spend between one to three hours in one household depending on how many patients they need to see or follow up. CHWs pointed out that they do not wish to make household members exhausted as a result of the visits. They, therefore, make sure that they do what brings them to the households and leave thereafter.

CHW supervisors are health facility staff assigned the role of supervising CHWs. They supervise CHWs’ activities and provide them with adequate coaching and support to ensure the quality of their work, including home visits and the accuracy of monthly reports. Providing ongoing, supportive supervision to CHWs is critical, and can improve motivation and engagement. CHWs are obliged to report to their supervisors and submit some of the deliverables, including monthly reports highlighting the activities performed. The main tools used on a daily basis as part of CHWs work are counter books, referrals form for sending patients to health facilities, pens, boxes for keeping referral forms, and weighing scale.

CHWs also stocked pregnant women’s information forms that they need to fill when visiting pregnant women at home as part of the requirement for another project in the district. These forms were submitted monthly to health facilities. Generally, counter books and pens were the main working tools used by CHWs to record all activities they undertake at community and health facilities. Mobile phones were also used for the purpose of communicating with clients and supervisors.

#### CHWs and managing referrals

As part of their routine activities and visiting households, CHWs provide referrals to patients to visit respective health facilities. It was noted that the decision to refer is based on the training guidelines for CHWs. However, it was noted that it is challenging for CHWs to remember all conditions that require referral when visiting households because they do not always carry guidelines with them. With this regard, the decision is based on the condition of the patient and illnesses that CHWs themselves feel to lack knowledge about and what they remember to have been taught during the trainings:

*“You may go to a household, and someone is sick, but looking at him, you feel like what he is suffering from is out of your knowledge. You ask yourself… mhhh! … what type of illness is this? … the best option then is to refer him to a higher level.*” (CHW, Kibaha TC).

Referral decisions were also based on recurrent illnesses that have not been treated after using medication from ADDO or using home remedies recommended by CHWs. More importantly, for danger signs and symptoms which CHWs feel like the patient needs higher care, a referral is always advised. For children under the age of five, reported conditions that require referrals to health facility as unveiled by CHWs include; high fever, convulsions, diarrhoea, bleeding of the umbilical cord and breathing complications. Among new-borns, pregnant and postpartum women, referral to health facility are offered when pregnant women feel severe pains in any part of the stomach, bleeding complications, leg swelling and constipation. Adolescents in need of family planning services, especially injections were also directed to health facilities.

The findings suggest that the majority of CHWs refer patients to health facilities; only a few referred the patient to ADDOs, reasons being that health facilities are a place where CHWs have been told to refer patients, but more importantly, CHWs feel like patients are likely to get all tests and diagnosis done at the health facility compared to drug outlets. As highlighted above, referrals to health facilities were done using a paper-based system. In case referral papers are out of stock or not available, oral referrals are provided and the respective patient when reaching the health facility has to report that he has been seen by a particular CHW who did not have a referral form.

This was necessary to facilitate prompt patient management in respective health facilities. The referral paper had a separate feedback paper to be returned to the CHW after the patient has been attended at the heath facility. This is a way of confirming that referrals have reached the intended destination. Tracking of referrals was reported to be challenging as not all referred patients who have been seen by the health provider at the health facility return the feedback paper. CHWs have to follow patients at their households to see whether or not they reached the health facility.

The other challenge with regard to referral is the fact that not all who are provided with referrals attend the health facility. The main challenge reported by the CHWs’ supervisor is that some CHWs do not submit their monthly reports on time because they reside far from health facilities, and they have to pay for their transport to reach their respective health facility each month for report submission.

#### ADDOs workflow procedures and challenges

ADDO dispensers reported to work between nine and thirteen hours a day. The need to open for longer hours as reported by dispensers is to cater for clients coming late from work or farms. ADDOs that are located in town reported to see between 30 and 70 patients per day, while those located in rural parts attend between 10 and 20 patients per day. Patients who come with prescriptions were attended for not more than four (4) minutes while those who present at the outlets to be assessed and managed take between 8 and 25 min.

The management of ADDOs at higher levels was reported to be done by the Government through the Pharmacy Council (PC) who frequently send supervisors to the outlets for inspecting medicines and cosmetics status, and assess whether the dispenser received ADDO training. Legal measures were reported to be taken by the PC for ADDOs who do not abide by the ADDO operation guidelines. At the district level, ADDOs received supervision from the District Pharmacist.

When asked how they keep records and documentation of drugs and patient’s information, ADDOs use drug register books provided by the Pharmacy Council to record patients’ potential diagnosis, medicine prescription and any referrals given to health facilities. At times, the Pharmacy Council does not supply these register books, and therefore documentation is done using any books available as narrated here:

*‘‘Nowadays they do not bring us drug registers; some are still using those provided before. But you can talk to the Pharmacist, if he has some in stock, he can give the register to you. I asked him and he gave me one, and I am using it.” (ADDO Dispenser, Kibaha DC)*.

On a monthly basis, ADDO dispensers analysed all patients’ treatment information for profit assessment and send specific child health indicator data to the Pharmacy Council by mobile phone. It was noted that reliability of child data that is sent to PC is questionable because the analysis of the data is done manually by respective counting cases, and therefore shared data may be vulnerable to counting errors. Other challenges as reported by dispensers include lack of refresher training, and referral refusal by some patients who demand to be provided with medication even when the respective condition requires referral to health facilities.

The study also intended to explore the confidence of ADDO dispensers in treatment provision. Thus, we asked them the question, ‘how confident are you in managing your clients’? Two ways of attending clients were observed: dispensing medicine based on the prescription; and dispensing at the outlet based on the dispenser’s assessment of the patient. Both options were reported common and the dispenser could not say which outnumbers the other. Dispensing through prescription had fewer challenges because what a dispenser needs to do is to dispense based on what the healthcare provider wrote in the prescription. The main challenge reported under this type of management is when what is prescribed is not in accordance with the symptoms of the patients as described here:

*“A patient may come with a prescription, when you look at it and the way the patient explains what he is suffering from, you immediately know that what is written in the prescription will not assist the patient. But because it is written by the doctor, what else can you do? You just give the patients what is written.”* (ADDO Dispenser, Kibaha TC).

Mixed views were noted with regard to the ability to dispense after assessment of the patients who visits the outlet. The study assessed three main indicators for managing clients: ability to identify more than one illness, competence in treatment provision and assurance that the medication provided will treat the condition. Majority of dispensers reported to try their best to identify illnesses when attending patients. This was done by asking for more than one illness the patient has faced, and the duration of symptoms, as described below.

*“When he says that he has a headache, there are follow-up questions that I will ask him. Maybe, “Do you also feel stomach ache? Where is the pain located?” This will assist me to determine if I am in a position to manage the clients by giving him medicine, or refer him to a health facility.”* (ADDO Dispenser, Kibaha DC).

Some dispensers expressed the challenges which they face in recognizing what the patient is suffering from, especially when the condition involves a number of symptoms. In such cases, dispensers advise patients to go for laboratory tests. ADDO dispensers reported to refer patients to health facilities either orally or by using referral forms. However, it was observed that many ADDOs do not use referral forms when referring because of the forms being out of stock, while other dispensers had made a significant number of copies which are kept at the outlets to be used for the same purpose. Referral forms were provided by the Pharmacy Council in 2007 through donor funding when the ADDO program was launched in the districts, an initiative that did not continue thereafter. Referral feedback from health facilities to ADDOs was either poor or not in existence.

A referral paper has a section that needs to be filled by the provider for the patient to return to the respective ADDO. In most cases, patients do not return such papers to ADDO, and therefore, the dispenser is not in a position to understand if the patient really arrived at the health facility as described below.

#### Health facility staff workflow procedures and challenges

Majority of dispensaries used the paper-based system of registering patients and documented their health records while health centres used both systems, paper based and electronic based. Paper based is done by registering all patients’ information from the facility registration book to the National Health Information System, abbreviated in Swahili as MTUHA (*Mfumo wa Taarifa za Uendeshaji Huduma za Afya)*. MTUHA was launched in Tanzania in 1993 and a second version in 1998. The system consists of a series of registers for primary data collection and secondary data books where information from the registers is totalled or used for calculation.

Similarly, electronic registration and documentation was done using a system known as Government of Tanzania Hospital Management Information System (GoT-HOMIS). GoT-HOMIS is an integrated Health Facility Electronic Management Systems (iHFeMS) in the health facility with the intention to improve the quality of operations while enhancing revenue collection, management of medicines, and other medical supplies. GoT-HOMIS enables patient registration, tracking and providing effective administration and control at the facility.

Furthermore, GoT-HOMIS supported the integration of functions for smooth patient movements within various services at the heath facility. The reported reason for using both systems is insufficient number of computers at all levels of providers within the facility necessary to fully implement the GoT-HOMIS system that leads to difficulties in tracking patients’ registration number, double dispensing, and double registration.

#### Referral handling in health facilities

Dispensaries received patients who come with or without referral forms referred by CHWs. Referred patients who cannot be managed at the levels of the dispensary were referred to the higher-level health centre. Besides, no formal system is existing to follow up patients who have received treatment at a health facility but required further follow-ups at the community level. What is done is to link the patients with the respective CHW or provide them with the phone number of the CHW in case assistance is needed in the community. On the other hand, it was revealed that CHWs are very active to follow up patients whom they have referred to health facilities as narrated here:

*“Oooh! CHWs are very proactive when it comes to follow up on their patients. When they refer a patient here, they will call to see how the patient is doing, and even when the patient goes back home, they will continue with the follow up there and give us updates.”* (CHW supervisor, Kibaha DC).

A big majority of health facility staff did not receive formal referrals from ADDOs, and only linked patients to ADDO when facilities are out of stock of medicines.

#### Coordination challenges among CHWs, ADDOs and health facilities

As already reported, the majority of CHWs provide referral to health facilities, some use referral papers and others not, others escort the patients to health facilities while others refer the patients while calling the respective health facility in charge, informing them about the referred patients so that they are well received. CHWs reported that it is challenging to understand whether or not the referrals provided have reached health facilities because there is no formal mechanism to track the referred patients.

Conversely, referrals from CHWs to ADDO were reported by few CHWs and done during weekends when health facilities are short of staff. Referrals from ADDOs to health facilities are done when patients are seriously sick and cannot be attended at ADDO. Referrals from health facilities to ADDO were done when health facilities are out of stock. At all these levels, there is no referral feedback mechanism to track patients and confirm whether they reached an intended destination. ADDO dispensers reported challenges when they receive patients with wrong prescriptions from the health facility, they have no mechanism to communicate this to the respective health facility and need to send the patient back to the health facility, something that patients don’t prefer. Some ADDO dispensers who were interviewed do not know the role of CHWs while few did not know whether CHWs exist in their setting. It was again reported by ADDO dispensers that at times it is challenging for them to refer patients to health facilities because people prefer to be treated at ADDO and avoid long waiting time at health facilities:

Challenges reported by dispensers on the part of the patient include the fact that patients who come with the prescription from the health facility may use that prescription more than once, and at different ADDOs to obtain medicine for themselves or relatives. It is challenging to know whether the prescription has been used or not because there is no patient record. The challenge of feedback mechanisms is well explained by a health facility staff:

*“Yes, you have reminded me that this is a challenge that we have as healthcare providers. I do not understand what this is, the child was brought here with diarrhoea and given medication. She was also tested for malaria, and it was negative. The following day she came complaining that the child is still very sick, and she decided to purchase antimalarial from the ADDO and start the dosage. I told her that the child not have malaria, but she said that she was worried and did not trust the malaria tests because the diarrhoea did not stop. If we had good communication with ADDO dispensers, this could not have happened. We should both speak same language, but the story continues. We could not manage the child here, so we gave her a referral. When she reached the hospital, she was again administered with another dose of antimalarial, and she came here showing me the two bottles of antimalarial, although different brands, but both were antimalarial.”* (Health facility staff, Kibaha DC).

### Technology literacy and perceptions towards the use of technology

Majority of CHWs used analogy mobile phones, and did not have enough experience using technology, particularly smartphones. More ADDO dispensers in Kibaha TC, than Kibaha DC owned smartphones, and were more experienced in using technology and smart phone features. Most health facility staff in both districts owned smartphones. Interestingly, all the participants expressed their interests to learn the use of technology for improving the quality of care.

The participants did not show any concern on Afya-Tek coming up with the use technology in the project, particularly in the use of fingerprint, palm and facial for data collection and storing communities’ health statistics. It was noted that communities are used to fingerprint registrations since such techniques have been used as requirements for national election exercise and registration of mobile phones. The participants, however, advised that the community first needed to be educated about the importance of using any digital devices before project implementation.

Internet connectivity and mobile phone networks were found to be strong in most areas with the exception of a few villages. In some of these villages, charging of mobile phones when Afya-Tek activities start was an anticipated challenge. The proposed option was to charge during the day at community places that offer solar energy services. All mobile phone companies had network coverage in the areas. The companies include Tigo, Vodacom, Airtel, with some being stronger than others in specific areas.

#### Perception on use and requirements for technology to link CHWs, ADDOs and health facilities

All participants and particularly CHWs, ADDO dispensers and health facility staff expressed the need for having a system that would link them and assist patients as they move through different places in seeking care.

CHWs in particular thought that the digital technology would simplify their work as there will not be any need for using household registers when visiting households and all data will be stored in the devices where it is safer. The reported challenge for using paper registers was associated with safety and storage, but was also challenging during monthly report generations where one has to go page by page. This was also supported by CHV supervisors who thought that the use of technology would enhance quick generation of reports to be accessed online and timely.

We probed more into the requirements CHWs would like to see in the technology. Many of them think that it is important that when they refer patients to health facilities or ADDO, they receive feedback that the referred patient has reached the intended destination. This was deemed very important by CHW as explained here:

*“Feedback will help us a lot, but also smooth our work. You may give a referral to a patient and may not be aware whether he/she went to the health facility, until you go for another round of household visits. Thus, if there are mechanisms for us to know whether the refereed cases have reached the destination, that would indeed be helpful for us.”* (CHW, Kibaha DC).

Likewise, ADDO dispensers expressed the need to communicate with CHWs and health facilities. They viewed the digital intervention as an innovative approach to assist communities as they struggle seeking healthcare. But they also see it as a tool that would simplify their work at the outlets. ADDO dispensers’ concerns on the requirements of digital technology is that once a referred person scans a fingerprint, as the dispenser, he should be able to see where the referral came from, so that in case of any concern he may call the person who made the referral. More importantly, ADDO dispensers are of the view that digital systems and scanning of patients would eliminate the challenge of most customers using prescriptions more than once, because at every point of healthcare patients’ history will be generated to allow a provider to assess previous dispensing.

CHMT teams in both councils thought that the digital intervention would provide reliable data on the burden of health problems facing women, children and adolescents. This was deemed crucial because people have different options for seeking care, but once all those options are harmonised with a digital platform, data on the burden of the problem will be reliable to inform future intervention areas. Decision support tools were also welcomed by both CHWs and ADDOs, provided that they are in line with what they were trained on. The main concern was for the decision support tools not to be very long to ensure that clients and patients are not exhausted and feel uncomfortable to continue answering.

Some participants expressed their concerns on the use of biometrics among adolescents. Since adolescents seek care secretly and prefer anonymity, they may pose a challenge when it comes to scanning their fingers, assuming that their treatment histories will be shared widely. In conversation with adolescents, however, this was not the case, the majority indicated their desire to scan fingerprints if education is provided on the reasons to do so.

All participants, including community members, expressed their interest to use technology in case it is intended to improve the quality of care; and most important for vulnerable groups in the community, women, children and adolescents.

## Discussion

The findings highlight the need for care coordination among CHWs, ADDOs and health facilities in order to improve the quality of healthcare to mothers, children and adolescents. In order to achieve this, the Afya-Tek digital intervention aims to improve the continuity of care and assist patients as they navigate through multiple points of care within the Tanzanian local health system. Particularly, the digital health intervention when designed, needs to take into account a variety of perceived challenges.

It is important noting that the Afya-Tek intervention was envisioned in 2015, based on a project supported by Management Sciences for Health which worked to strengthen the quality of healthcare in Kibaha, Tanzania by improving linkages among three actors within the primary healthcare system: Community Health Workers (CHWs), Accredited Drug Dispensing Outlets (ADDOs), and primary health facilities [5]. By improving linkages and strengthening referral coordination, the 2015 project hoped to improve access to healthcare throughout the continuum of care as clients navigate various levels within the health system.

While this prior project showed promising results and acceptability among health workers to better integrate services, significant challenges in standardising decision-making processes, share medical history and uniquely identify clients throughout the system prevented the project from demonstrating a truly integrated continuum of care [5]. The Afya-Tek intervention, thus, leverages the learnings from the mentioned study, and thereby developed the concept of a co-created, human-centred digital health intervention which coordinates care along the three health system actors (CHWs, ADDOs and Health Facilities) in order to support clients as they navigate through multiple points of care.

Key findings from this formative research indicate that there are delays in communication and feedback between CHWs and their supervisors currently, which were contributed to by difficulties in physical meetings in health facilities. Across sub-Saharan Africa, proper communication between CHWs and their supervisors remains a challenge. This finding is supported by the fact that delays in accessing information pertaining to health activities is a stumbling block towards the realization of real-time information flow in the healthcare system [6, 13]. A study conducted in Zambia by Biemba and colleagues [13] focused on a digital platform that was developed to tackle communication barriers and improve real-time communication between CHWs and supervisors and shed light on the need for Afya-Tek to take this into consideration.

Bridging these communication gaps between CHWs and their supervisors is critical for an effective flow of information between CHWs and their supervisors, and highlights the value of real-time managed information flow, especially in low resource countries. Through the use of Afya-Tek’s co-created technology and digital systems, CHWs supervisors should be able to frequently assess the performance of the CHWs, receive monthly reports in time, as well as provide timely feedback to CHWs.

Study findings also indicate that both CHWs and ADDO dispensers experience some challenges in reaching an informed decision about patient diagnosis, especially when patient experiences multiple symptoms. A study carried out by Dillip and colleagues in 2017, noticed the poor, or rather lack of informed decision-making about patient diagnosis among the two aforementioned healthcare actors operating at the community level. The study further highlighted that the lack of properly informed decision has complicated proper referral arrangements and continuity of care. In this study too, referral feedback was also a key reported concern among both CHWs and ADDOs. CHWs, for instance, expressed the need to ensure that the technology gives them feedback on the patients they refer to either ADDOs or health facilities – this is something that will increase work efficiency and motivation, but also contribute to ensuring prompt care seeking.

The design of a digital intervention should ensure timely, respected and reliable care at all points of care provision. As such, the Afya-Tek decision support tool will be designed with the intention to simplify work and guide the provision of appropriate care and referrals for each of these health provider groups. Additionally, at the ADDOs there is a shortage of drug registers and repeated multiple usages of a single prescription by the community, which the ADDOs cannot currently keep track of. Whilst there is a dearth of information on the way ADDOs operate due to a scarcity of studies undertaken, [5] highlight this lack of proper systematic mechanism to record and track patient’s history and information. The digitization of working tools and referrals at ADDOs was viewed positively and seen as a solution to the challenge of drug register shortages as well as that of clients also using a prescription more than once.

From a community perspective, pregnant women, children and adolescent groups were considered vulnerable by the majority of the participants. This was also supported by both Pharmacy Council and CHMT teams in both district councils. The state of vulnerability regarding the health of women, children and adolescents is hard to ignore in the current effort to attain universal health coverage. Literature continues to highlight the risks that women, children and adolescents are facing, especially in low-income countries where digital solutions remain challenging [14].

The lack of proper mechanisms to tackle these health challenges has been a reason for increased persistence of health challenges caused by untimely access of information [5, 6, 15]. This suggests the need to generate a digitised solution that can ensure timely and reliable information. Thus Afya-Tek’s digital intervention has prospects for generating reliable data on the burden of disease among pregnant women, children and adolescents.

In particular, adolescents were reported to start sexual practices at a very young age. Because of privacy reasons, most adolescents preferred seeking care at ADDOs. While adolescents form a socially important segment of the population [16], numerous and studies explain how reproductive health amongst this group remains one of the unspoken aspects of health in many LMICs. Prevailing socio-cultural norms and values continue to maintain privacy barriers and restrict public discussion on most issues related to adolescent reproductive health. In his study on adolescents in the southern part of Tanzania (Mtwara region), [17] unveils how adolescent reproductive health is embedded in social and cultural value.

There is a high degree of privacy in discussing adolescent health status, to the extent that neither community nor adolescents themselves can discuss it publicly. Arguing on the adverse effect of this privacy, [18] assert that the majority of adolescents are exposed to various health risks and challenges that remain to be tackled. For instance, abortion is restricted by laws in Tanzania, which in recent times is more widely practiced among girls, thus rendering increased deaths and other health challenges [18, 19]. By taking these privacy concerns into account, the Afya-Tek intervention could, therefore, ensure that ADDOs are equipped with necessary skills to provide appropriate care among this age group.

There remain a few structural and systemic concerns for the design of such a digital health intervention in such contexts. A few villages did not have reliable electricity and network connectivity. Access to network connection and electricity are two challenges facing the communication arena in LMICs [20–22]. For instance, irregular power supply and poor internet access were identified as two challenges impacting the limited success of an on-line training programme in Ethiopia [23]. Meanwhile, in Tanzania the percentage and number of mobile phones, as well as the internet subscribers [24], has increased rapidly.

According to the Tanzania Communications Regulatory Authorities (TCRA) report of 2020, the current data indicate that in the end of September 2020, the number of mobile internet users in the country stood at 27,900,069 compared to 26,832,089 recorded at the first quarter in March 2020. With the growing use of mobile and internet technology, it is imperative to also consider the structural provisions of mobile networks, and electricity and power, when designing digital interventions. As such the design of the intervention needs to recognize the status of network connection and electric power to support the design and implementation of the digital intervention. Finally, the design of the intervention also needs to take into consideration the use of offline applications to ensure activity continuation.

Findings from this formative research are currently being used to inform the subsequent phases of the Afya-Tek project in the co-development of the digital health intervention and implementation with all relevant stakeholders in the district councils. This will help ensure that the solution is designed specifically to meet the needs of those who will be using the technology and the larger health system. As digital technologies became ubiquitous in recent years [22, 25, 26], there is a large body of literature and studies on the growing trend to integrate digital technologies in the milieu of healthcare systems and delivery.

In today’s digitised world, digital technologies are considered essential tools for health promotion and solutions to healthcare delivery challenges [22, 26, 27]. While current scientific literature generally acknowledges the value of digital technologies as a health system strengthening tool [28], there is an argument that technologies in and of themselves cannot offer and enable new ways of coordinating and ensuring the quality of care [15]. This highlights the importance of how digital health interventions are designed in order to effectively coordinate care, tackle health challenges, as well as ensure continuum and quality of care [29].

As such, human-centred design has sparked growing interest as a mechanism to ensure inclusiveness, stakeholders’ engagement and foster an iterative approach that engulfs the relationship between design and implementation [25, 29–31]. The usefulness of human-centred design in improving outcomes is acknowledged in several studies on health interventions and other development interventions or programs across various disciplines. Reflecting their own experiences with over seventy digital health initiatives, [25, 29], are of the view that human-centred design is a practical way of accompanying health workers and communities in their struggles for the quality of health and equity.

The ssuccess of the human-centred digital health interventions has been shown across evaluation studies conducted by Holst and colleagues (2020) in South Africa, Uganda and Ghana. They highlight how outcomes of digital interventions in the mentioned countries were achieved due to the use of human-cantered design as a non-discriminating approach in co-designing digital interventions [27]. Similarly, Huang and colleagues [25] observed the fact that, in Uganda, despite severe resource constraints, through the use of human-centred design the digital system was successful in realising its outcomes.

The same observation was realised in China where Huang and colleagues suggest that the reason for success lays not on the availability of the resources, but rather the use of a human-centred approach in designing the intervention. Thus, the Afya-Tek project recognized the importance of enabling new ways of coordinating, decentralizing and expanding the quality of care [15]. Prior to designing the intervention, Afya-Tek realized the need to use a human-centred design as an approach to make the desired goals of the intervention successful. Both achievements for interventions using human-centred design and failure of the interventions ignoring the use of human-centred design as postulated by literature and studies, stress the validity of Afya-Tek in firmly incorporating the views of users and different stakeholders prior to designing the digital solutions.

With these findings, it highlights the importance of conducting formative research prior to designing the interventions. In this formative research, the findings have ultimately informed the co-design of the Afya-Tek project and the way it will impact the health system locally and globally. Follow-up publications (in progress) will share how the findings of the formative research have been used in the co-design of the Afya-Tek intervention.

## Conclusion

This paper presents the findings of the formative research conducted prior to designing and implementation of the digital health intervention. Our study emphasizes important aspects about health among different social groups, that is, mothers, children and adolescents based on objectives guided the study. Firstly, it explains the burden of diseases and how people navigate through multiple stations of care to remedy their health status. Secondly, it accentuates the work flow procedures and challenges facing healthcare actors including use of paper-based system for documentation and referral arrangement, poor coordination and fragmentation of care. Thirdly, it describes adolescent health aspects and health seeking complications. Lastly, it delineates people’s perceptions on the use of digital technology as a way of transforming healthcare delivery and the way they envision the designing of the digital health intervention.

From the findings, our study considers the value of digital technologies in the milieu of healthcare delivery as a mechanism for waging war against health burdens and challenges. In terms of the broader relevance, the findings from the formative research inform project designers to understand among others; health burdens and challenges associated with non-use of digital platforms. Given the current trend of digital platforms, the present study offers a significant insight to consider in a co-design and implementation of the digital health intervention prior to its implementation.

At the same time, the findings highlight the centrality of human-centred approach (as envisioned by people in the community of study) in designing digital health interventions which among others, stresses the need to involve local communities and health system actors. This ensures that digital interventions are closely aligned to the local needs as mechanisms to improve quality of healthcare locally and globally.

## Data Availability

Copies of the interview guides, transcripts and audio data are available from the first author.

## List of abbreviations

ADDO: Accredited Drug Dispensing Outlet
CHMT: Council Health Management Team
CHWs: Community Health Workers
GoT: HOMIS- Government of Tanzania-Hospital Management Information System
HIV: Human Immunodeficiency Virus
iHFeMS: Integrating Health Facilities Electronic Management
PC: Pharmacy Council
PO-RALG: President’s Office - Regional Administration and Local Government
